# Screening of m6A-associated ferroptosis-related genes in atherosclerosis based on WGCNA

**DOI:** 10.3389/fcvm.2024.1469805

**Published:** 2024-10-28

**Authors:** Meiling Jiang, Weidong Zhao, Liyong Wu, Guofu Zhu

**Affiliations:** Cardiology Department, The Second Affiliated Hospital of Kunming Medical University, Kunming, Yunnan, China

**Keywords:** m6A, ferroptosis, m6A-Ferr-related signature genes, WGCNA, immune infiltration

## Abstract

**Background:**

N6-methyladenosine (m6A) has been shown to mediate ferroptosis but its role in atherosclerosis (AS) is unclear.

**Methods:**

Differentially expressed m6A-associated ferroptosis-related genes (DE-m6A-Ferr-RGs) were obtained using differential expression analysis and Pearson correlation analysis. Weighted gene co-expression network analysis (WGCNA) was also performed. The intersection of the module genes and the DE-m6A-Ferr-RGs were recorded as candidate m6A-Ferr-related signature genes. Finally, the m6A-Ferr-related signature genes were screened using least absolute shrinkage and selection operator (LASSO) analysis. Expression validation, receiver operating characteristic ( mapping, and immune correlation analysis were also performed based on the m6A-Ferr-related signature genes. The expression of m6A-Ferr-related signature genes was further validated using a real-time polymerase chain reaction (RT-qPCR).

**Results:**

In total, 6,167 differentially expressed genes were intersected with 24 m6A- and 259 ferroptosis-related genes, respectively, resulting in 113 DE-m6A-Ferr-RGs obtained using Pearson’s correlation analysis. The module genes obtained from the WGCNA and the 113 DE-m6A-Ferr-RGs were intersected to obtain 48 candidate m6A-Ferr-related signature genes. LASSO analysis was performed and six m6A-Ferr-related signature genes were screened. In addition, the area under the curve values of all six m6A-Ferr-related signature genes were greater than 0.7, indicating that they had potential diagnostic value. Furthermore, the RT-qPCR results revealed that the expression of *SLC3A2*, *NOX4*, and *CDO1* was consistent with the transcriptome level. Moreover, there was a significant difference in two types of immune cells between the AS and control groups. Naive B cells, CD8+ T cells, regulatory T cells, and activated natural killer cells were positively correlated with *CDO1* and *NOX4* but negatively correlated with *ATG7*, *CYBB*, and *SLC3A2*.

**Conclusion:**

In total, three m6A-Ferr-related signature genes (*NOX4*, *CDO1*, and *SLC3A2*) were obtained through a series of bioinformatics analyses and an RT-qPCR.

## 1 Introduction

Atherosclerosis (AS) is a common pathological foundation for numerous cardiovascular diseases (CVD) (1). AS is characterized by a disorder in lipid metabolism, smooth muscle hyperplasia, endothelial dysfunction, apoptosis, necrosis, inflammation, and the formation of foam cells and lipid plaques (2). In addition, increasing evidence indicates that epigenetic modifications are associated with the onset and progression of AS (3). N6-methyladenosine (m6A) methylation modification is one of the most prevalent epigenetic alterations in eukaryotic RNA. Dysregulation of m6A modification levels occurs in various pathological and physiological processes, including AS (4). In recent years, researchers have confirmed that the occurrence and development of AS are closely linked to m6A RNA methylation (5). For example, m6A methyltransferase *METTL3* promotes angiogenesis and atherosclerosis by upregulating the *JAK2/STAT3* pathway through the m6A reader IGF2BP1 (4). The lack of *METTL3* in macrophages inhibits the formation of AS plaque induced by hyperlipidemia (6). Ferroptosis, a newly discovered form of regulatory cell death, is characterized by iron-dependent cell death and excessive lipid peroxidation. Recent studies have indicated that ferroptosis can promote the progression of AS through iron-dependent lipid peroxidation (7). Various pathological and physiological events related to AS, including disorders in lipid and iron metabolism, oxidative stress, oxidized low-density lipoprotein (Ox-LDL)–induced vascular endothelial cell injury, and inflammatory reactions, are associated with ferroptosis (8).

Furthermore, key regulatory factors of ferroptosis have been found to exhibit abnormal levels of m6A under different pathological conditions (9). Increasing evidence suggests that m6A modification and m6 regulatory factors play a crucial role in regulating cell susceptibility to ferroptosis (10). In recent years, numerous studies have demonstrated that m6A modification plays a role in the regulation of ferroptosis and impacts the progression of diseases including cancer (11), chronic obstructive pulmonary disease (COPD) (12), liver fibrosis (13), and aortic dissection (14). The regulatory factors of m6A modification combined with ferroptosis-related genes can potentially serve as diagnostic or prognostic markers for human tumors (15). However, the association between m6A-modified ferroptosis and AS has not been thoroughly elucidated.

Therefore, we conducted a study that aimed to identify m6A-related ferroptosis biomarkers during the occurrence and development of AS. We screened for m6A-related ferroptosis diagnostic genes using weighted gene co-expression network analysis (WGCNA), screened for m6A-Ferr-related signature genes using least absolute shrinkage and selection operator (LASSO) regression analysis, and developed a diagnostic risk model for AS that aimed to reveal m6A-related ferroptosis biomarkers in AS.

## 2 Materials and methods

### 2.1 Data sources

AS-related expression profile data were obtained from the Gene Expression Omnibus (GEO) database (https://www.ncbi.nlm.nih.gov/geo/). The GSE43292 dataset (arterial tissues from 32 controls and 32 AS patients) was used as the training set and the GSE100927 dataset (arterial tissues from 12 controls and 29 AS patients) was used as the validation set. In total, 259 ferroptosis-related genes (Ferr-RGs) were obtained from the FerrDb database (http://www.zhounan.org/ferrdb/current/) (16) (Supplementary Table S1) and 24 m6A regulators were obtained from the literature (17) (Supplementary Table S2).

### 2.2 Differential expression analysis

To identify the differentially expressed genes (DEGs) between different samples, the data in GSE43292 were analyzed for DEGs associated with AS using the R language “limma” package (version 3.50.1) (18) with an adjusted *p*-value (*p*_adj_) <0.01 as a screening criterion. Volcano and heat maps were plotted using “ggplot2” (version 3.3.5) and “pheatmap” (version 1.0.12), respectively.

### 2.3 Correlation calculation of differentially expressed m6A-associated genes and differentially expressed ferroptosis-related genes

The intersection of DEGs with m6A-related genes (m6A-RGs) was taken to obtain the differentially expressed m6A-associated genes (DE-m6A-RGs), using Venn (version 1.11) (19) to create the Venn diagrams. Similarly, the differentially expressed ferroptosis-related genes (DE-Ferr-RGs) were obtained. Pearson’s correlation was calculated between these genes. Genes that met the criteria (*p* < 0.001 and |*R*|>0.4) were recognized as differentially expressed m6A-associated ferroptosis-related genes (DE-m6A-Ferr-RGs), and the results of the calculation were visualized in a heat map.

### 2.4 Weighted gene co-expression network analysis

To identify genes associated with different traits, WGCNA (version 1.70.3) (20) was performed on the samples from GSE43292. A hierarchical clustering tree was first constructed for all the samples (*N* = 64), and a sample dendrogram and a trait (AS vs. control) heat map were constructed. Next, the appropriate soft threshold was selected based on the near-scale-free topological criteria to construct gene modules. The modules were then gathered according to the criteria of the dynamic tree-cut algorithm. The correlations of each gene module with traits (control and AS) were calculated, the conditions for selecting key modules were: non-gray modules, *p*-values <0.05, and |cor| >0.5. Finally, module membership (MM) and gene significance (GS) screening were performed, and the key module genes were screened using the criteria of |GS| >0.4 and |MM| >0.8, respectively.

### 2.5 Functional enrichment analysis

The DE-m6A-Ferr-RGs were intersected with the key module genes to obtain candidate m6A-Ferr-related signature genes. To investigate the biological pathways involving the candidate m6A-Ferr-related signature genes, the Gene Ontology (GO) functional enrichment and Kyoto Encyclopedia of Genes and Genomes (KEGG) pathway analyses of the candidate m6A-Ferr-related signature genes were performed using the “clusterprofiler” package (version 4.2.2) (21).

### 2.6 LASSO algorithm model

To further identify m6A-Ferr-related signature genes, LASSO analysis (22) of the candidate m6A-Ferr-related signature genes was performed using glmnet (version 4.1.2), with the “family” parameter set as “binomial” and the cross-validation parameter “nfolds” adjusted to 10 to obtain the cross-validated error maps and gene coefficient maps. Thus, the m6A-Ferr-related signature genes were finally obtained. Subsequently, expression analysis of the m6A-Ferr-related signature genes was implemented in the GSE43292 and GSE100927 datasets, respectively. Receiver operating characteristic (ROC) curves were drawn using the pROC package (version 2.3.0) (23) to evaluate the diagnostic value of the m6A-Ferr-related signature genes. The larger the area under the curve (AUC), the higher the accuracy. The R package “rms” (version 6.2-0) (24) was used to construct the nomogram to predict the incidence of AS. Calibration curves were plotted using “regplot” (version 1.1) (25). Decision curves (DCA) was also plotted using the “rmda” package.

### 2.7 Gene Set enrichment analysis

To find the significant pathways for the m6A-Ferr-related signature genes, single-gene gene set enrichment analysis (GSEA) (26) was performed. The filtering criteria were |NES| >1, *p*_adj_ <0.05, and *q*-value <0.25.

### 2.8 Immuno-infiltration analysis

To investigate whether there were differences in immune cells between the atherosclerosis and control groups, we used the false discovery rate (FDR) correction to calculate the proportion of 22 kinds of immune cells in the AS samples. Heat maps and boxplots were drawn for visualization. Lollipop mapping of the 22 immune cells and m6A-Ferr-related signature genes were plotted to show the correlation analysis result.

### 2.9 Expression validation of m6A-Ferr-related signature genes

The relative RNA expression level genes were quantified by a real-time polymerase chain reaction procedure. Thus, 12 frozen tissue samples were obtained from the College of Forensic Medicine, Kunming Medical University, of which samples 1–5 were the control group and 6–12 were the AS group. This study was approved by the Medical Ethics Committee of the Second Affiliated Hospital of Kunming Medical University in China. All patients had signed an informed consent form. The expression of m6A-Ferr-related signature genes was further validated via RT-qPCR. The total RNA of the 12 samples was extracted using TRIzol (Ambion, Austin, USA) according to the manufacturer's instructions. Reverse transcription of the total RNA to cDNA was carried out using a SureScript-First-strand-cDNA-synthesis-kit (Servicebio, Wuhan, China) based on the manufacturer's instructions. RT-qPCR was performed utilizing the 2xUniversal Blue SYBR Green qPCR Master Mix (Servicebio, Wuhan, China). The primer sequences for the PCR are shown in Supplementary Table S3. *GAPDH* was used as an internal reference gene. The 2^−ΔΔCt^ method was utilized to calculate the expression of key genes (27).

### 2.10 Statistical analysis

All bioinformatics analyses were undertaken in R language. The rank sum test was employed to contrast the data from different groups. *p* < 0.05 was considered to represent a significant difference.

## 3 Results

### 3.1 DE-m6A-Ferr-RGs in the AS samples

**Figure 1:**
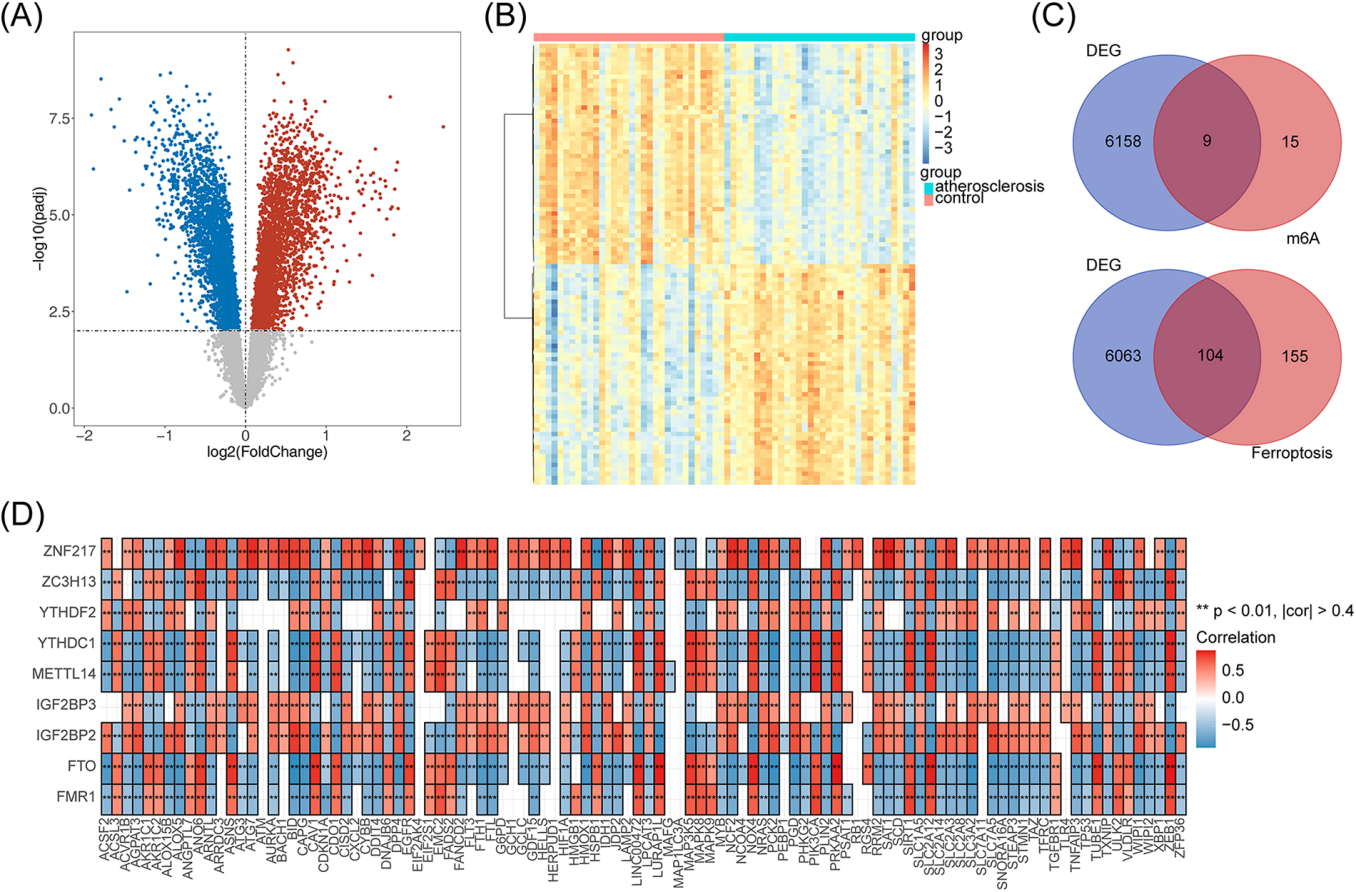
The DE-m6A-Ferr-RGs in the AS samples according to the differential analysis. **(A)** Volcano map of gene expression in the AS vs. control samples. Blue represents downregulated genes, red represents upregulated genes, and gray represents genes with no significant differential expression. **(B)** Heatmap showing differential gene expression in the AS vs. control samples. **(C)** Venn diagrams of DEGs with m6A-RGs and Ferr-RGs that result in ferroptosis. **(D)** The DE-m6A-RGs and DE-Ferr-RGs correlation heat map.

The differential analysis obtained a total of 6,167 DEGs in the AS and control samples from the GSE43292 dataset, of which 3,112 genes were upregulated and 3,055 genes were downregulated in the AS samples (Figure 1A). The top 100 upregulated and downregulated genes are presented in a heat map (Figure 1B). The intersection of the DEGs with 24 m6A-RGs and 259 Ferr-RGs resulted in nine DE-m6A-RGs and 104 DE-Ferr-RGs, respectively (Figure 1C). The results of the correlation between the DE-m6A-RGs and the DE-Ferr-RGs genes are shown in Figure 1D. In total, 113 genes satisfied *p* <0.001 and |*R*| >0.4 and were considered as DE-m6A-Ferr-RGs for subsequent analysis.

### 3.2 Screening for candidate m6A-Ferr-related signature genes

**Figure 2:**
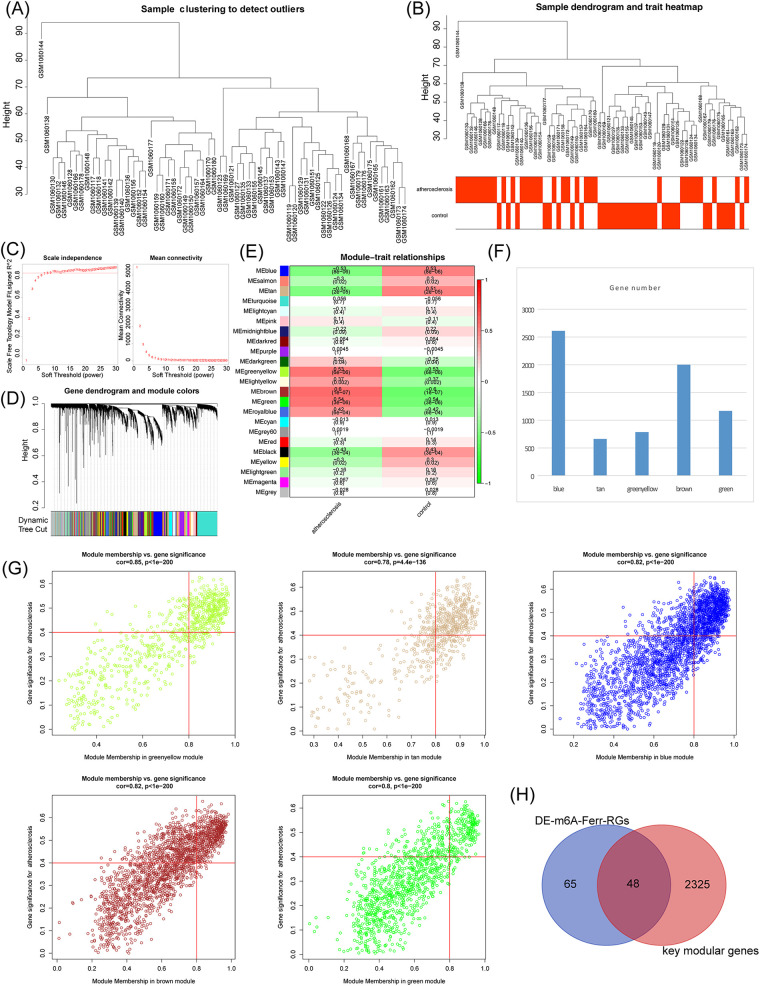
Screening of candidate m6A-Ferr-related signature genes by WGCNA. **(A)** Clustering of samples in the GSE43292 dataset. **(B)** Merged data sample clustering and phenotypic information. **(C)** Distribution of scale-free soft threshold values. **(D)** Tree diagram depicting module clustering. **(E)** Heatmap showing the correlations between modules and clinical traits. **(F)** Number of genes in each module. **(G)** Scatter plot demonstrating the relationship between key modules in MM and GS. **(H)** Venn diagram of DE-m6A-Ferr-RGs and key modular genes.

WGCNA was performed in the GSE43292 dataset. The hierarchical clustering tree results, as shown in Figure 2A, showed no significant outlier samples, so all samples were used for the subsequent analysis. The clustering and trait heat map of the AS and control samples is shown in Figure 2B. As shown in Figure 2C, nine with the best soft thresholds were chosen to construct the gene modules, and 23 gene modules were obtained, among which the gray module was non-sense (Figure 2D). The heat map shows the correlation between the 23 gene modules and the AS and control groups (Figure 2E). The key modules associated with AS were the blue, tan, green, yellow, brown, and green modules according to a *p*-value of <0.05 and |cor| of >0.5 (Figure 2F). The key module genes were screened using the criteria of |GS| >0.4 and |MM| >0.8, respectively, and a total of 2,373 module genes were obtained (Figure 2G). Finally, the DE-m6A-Ferr-RGs were intersected with the key module genes to obtain 48 candidate m6A-Ferr-related signature genes (Figure 2H).

### 3.3 Enrichment analysis of the candidate m6A-Ferr-related signature genes

**Figure 3:**
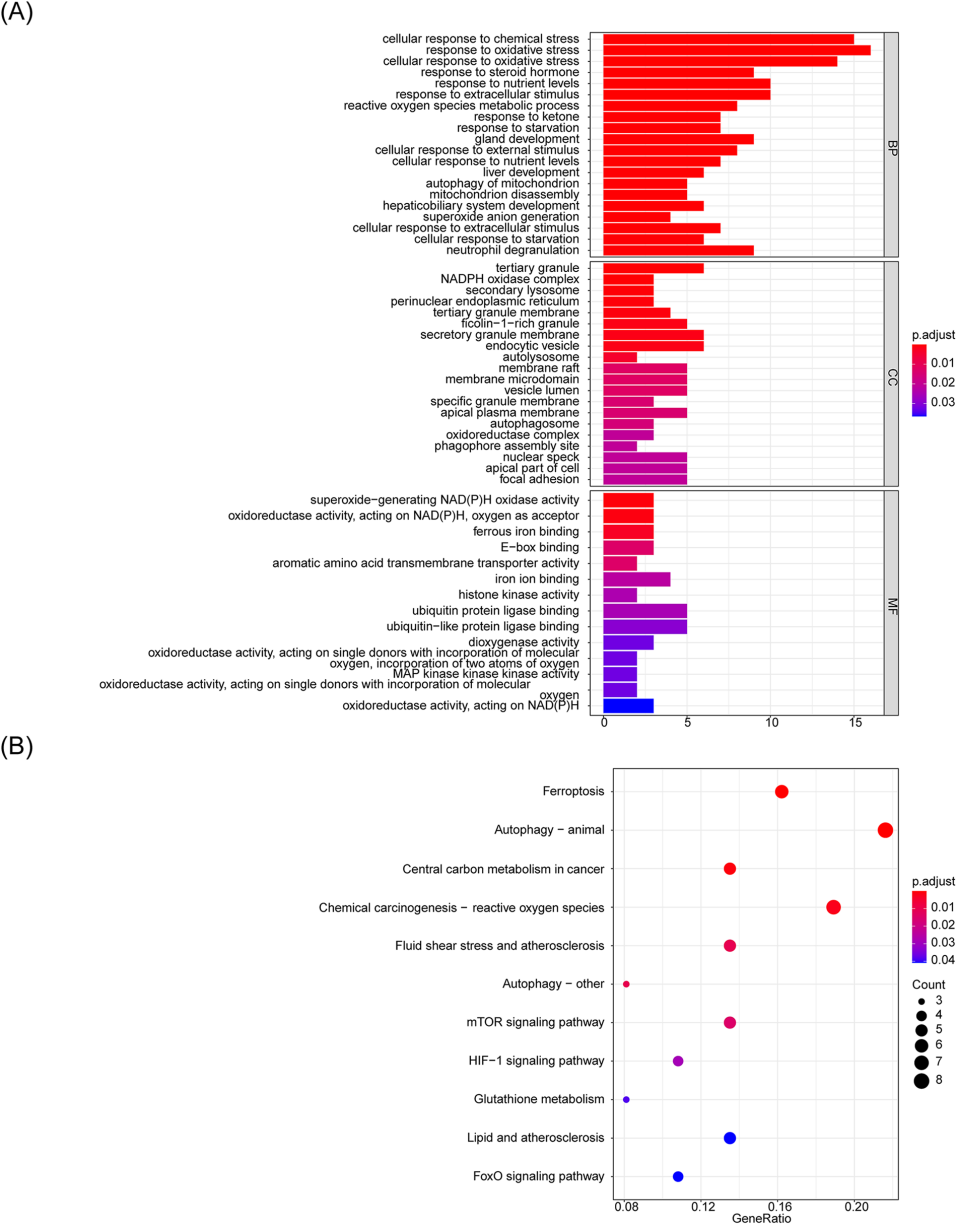
GO functional enrichment analysis of candidate m6A-Ferr-related signature genes. **(A)** Bar graph representing the top 10 Gene Ontology enrichments. **(B)** Bubble chart illustrating KEGG pathway enrichments.

To determine the potential biological roles of the selected candidate m6A-Ferr-related signature genes, we performed enrichment analyses. The GO functional enrichment analysis resulted in a total of 295 items, including 250 biological process (BP) items (cellular response to chemical stress, response to oxidative stress, neutrophil degranulation, etc.), 31 cellular components items (tertiary granule, endocytic vesicles, membrane rafts, etc.), and 14 molecular functions items (ubiquitin protein ligase binding, ferrous iron binding, dioxygenase activity, etc.) (Supplementary Table S4), and Figure 3A shows the top 10 ranked items under each GO classification. The results of the KEGG pathway analysis showed that a total of 11 pathways were enriched, and the enriched pathways [ferroptosis, autophagy-animal, chemical carcinogenesis-reactive oxygen species (ROS), etc.] are shown in Figure 3B.

### 3.4 Screening for m6A-Ferr-related signature genes

**Figure 4:**
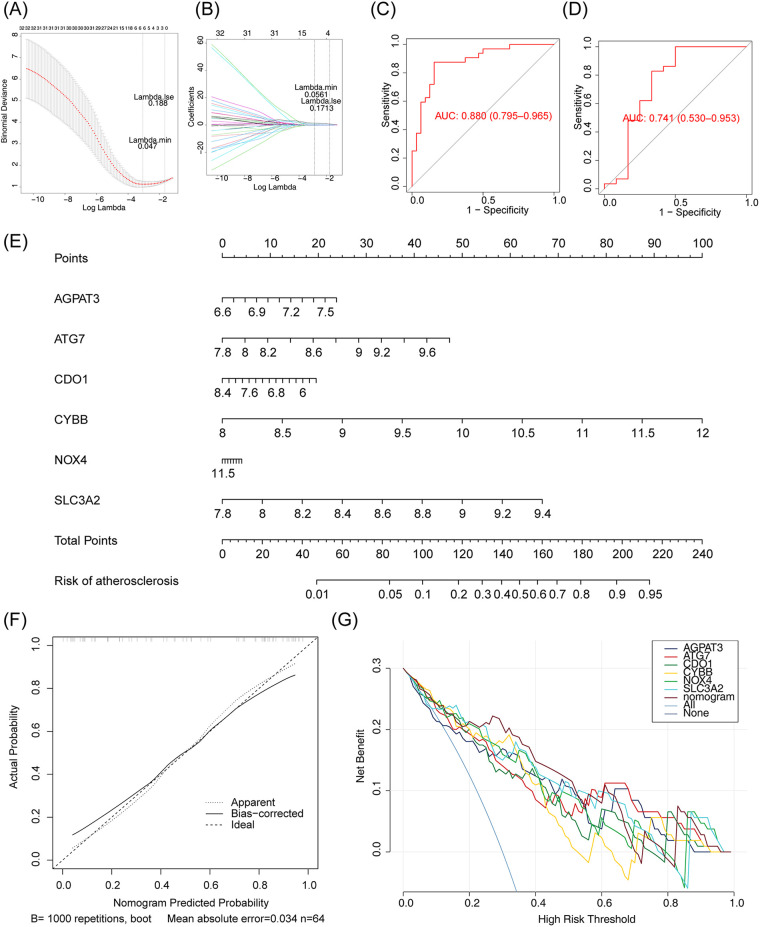
Screening m6A-ferr-related signature gene using LASSO analysis. **(A)** LASSO logistic regression coefficient penalty plot. **(B)** LASSO logic coefficient penalty diagram. **(C)** LASSO model ROC curve in GSE43292 dataset. **(D)** LASSO model ROC curve in GSE100927 dataset. **(E)** Nomogram predicting the incidence rate. **(F)** Column chart correction curve. **(G)** Decision curve for the LASSO model.

Screening for m6A-Ferr-related signature genes was conducted using LASSO regression analysis. The LASSO analysis screened six genes (*AGPAT3*, *NOX4*, *CDO1*, *CYBB*, *ATG7*, and *SLC3A2*) that were recorded as m6A-Ferr-related signature genes (Figures 4A,B). ROC curves of the LASSO model with AUC values of 0.880 and 0.741 in the GSE43292 dataset and the GSE100927 dataset, respectively (Figures 4C,D), indicated that the model performed well. Thus, a nomogram was plotted based on the six genes. The adjusted C-index of the nomogram was 0.835, indicating that the selection of the genes was appropriate (Figure 4E). The calibration and decision curves confirmed the above conclusion (Figures 4F,G).

### 3.5 Analysis and verification of m6A-Ferr-related signature genes

**Figure 5:**
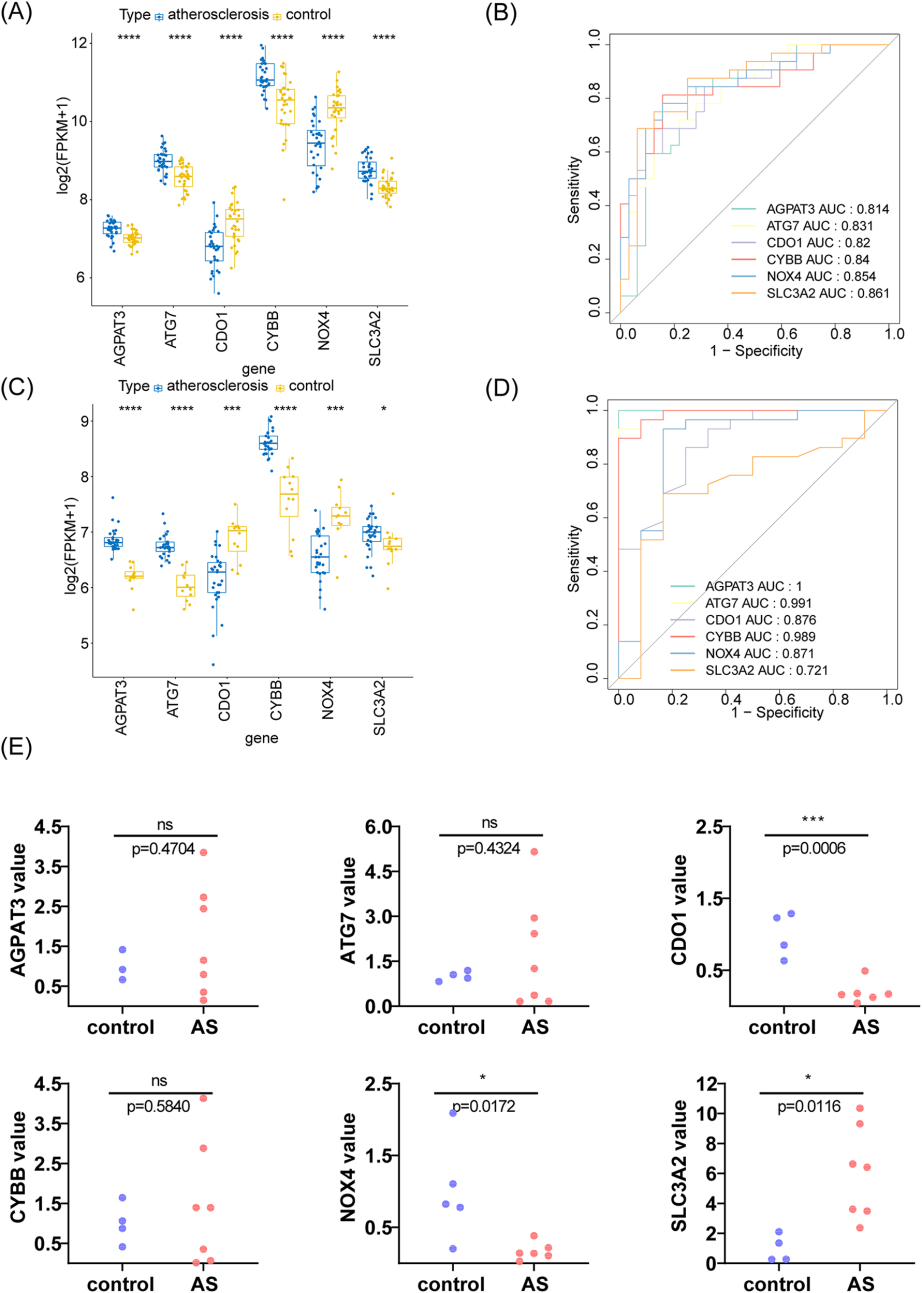
Analysis and verification of m6A-Ferr-related signature genes using boxplot analysis, ROC curve, and an RT-PCR. **(A)** Boxplot analysis using the rank sum test for the GSE43292 dataset. **(B)** ROC curve for the GSE43292 dataset. **(C)** Box-and-whisker plot analysis using the rank sum test for the GSE100927 dataset. **(D)** ROC curve for the GSE100927 dataset. **(E)** Expression of the six genes in the AS and control samples using an RT-PCR.

The expression of the m6A-Ferr-related signature genes in the AS and control samples from GSE43292 is shown in Figure 5A, in which *AGPAT3*, *ATG7*, *CYBB*, and *SLC3A2* were highly expressed and *NOX4* and *CDO1* were slightly expressed in AS group. Furthermore, the expression of the m6A-Ferr-related signature genes was verified in GSE100927. As shown in Figure 5C, the expression trend of the genes in the GSE100927 dataset was completely consistent with the GSE43292 dataset. In addition, in the GSE43292 and GSE100927 datasets, the AUC values of all six m6A-Ferr-related signature genes were greater than 0.7, indicating that they could distinguish AS patients from the control samples and had potential diagnostic value (Figures 5B,D). We collected 12 histological samples for RT-qPCR validation, and the detailed disease information of the corresponding patient samples is presented in Supplementary Table S5. The RT-qPCR results revealed that the expression of *SLC3A2*, *NOX4*, and *CDO1* was consistent with the transcriptome level. The expression trend of *AGPAT3*, *CYBB*, and *ATG7* was consistent but there was no difference between the AS and control groups (Figure 5E).

### 3.6 Single-gene GSEA of m6A-Ferr-related signature genes

**Figure 6:**
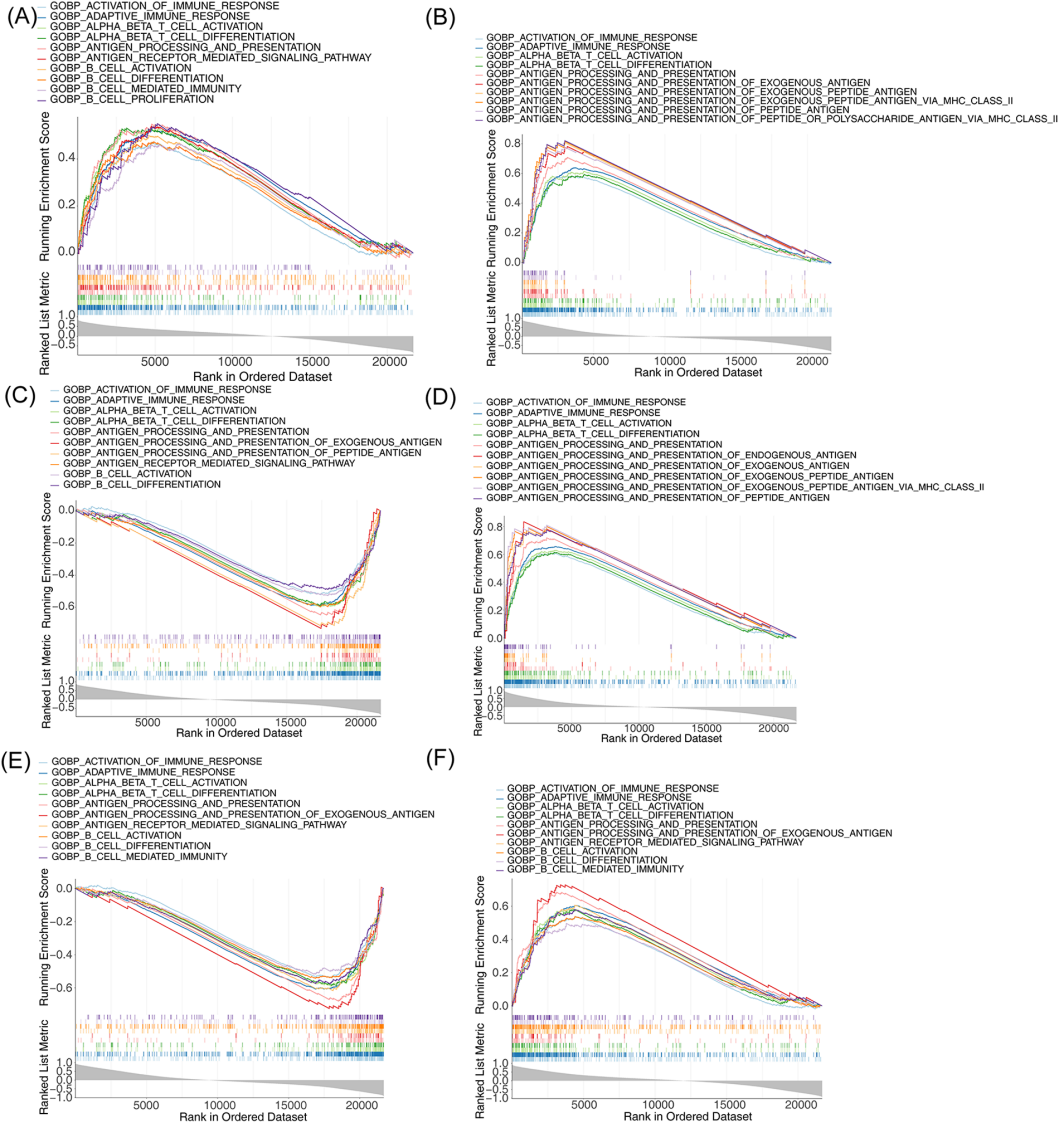
Single-gene GSEA for m6A-Ferr-related signature genes. **(A)** Results of GO enrichment analysis for *AGPAT3* using the single-gene GSEA method (top 10). **(B)** GO enrichment results for *ATG7* using the single-gene GSEA method. **(C)** GO enrichment results for *CDO1* using the single-gene GSEA method. **(D)** GO enrichment results for *CYBB* using the single-gene GSEA method. **(E)** GO enrichment results for *NOX4* using the single-gene GSEA method. **(F)** GO enrichment results for *SLC3A2* using the single-gene GSEA method.

**Figure 7:**
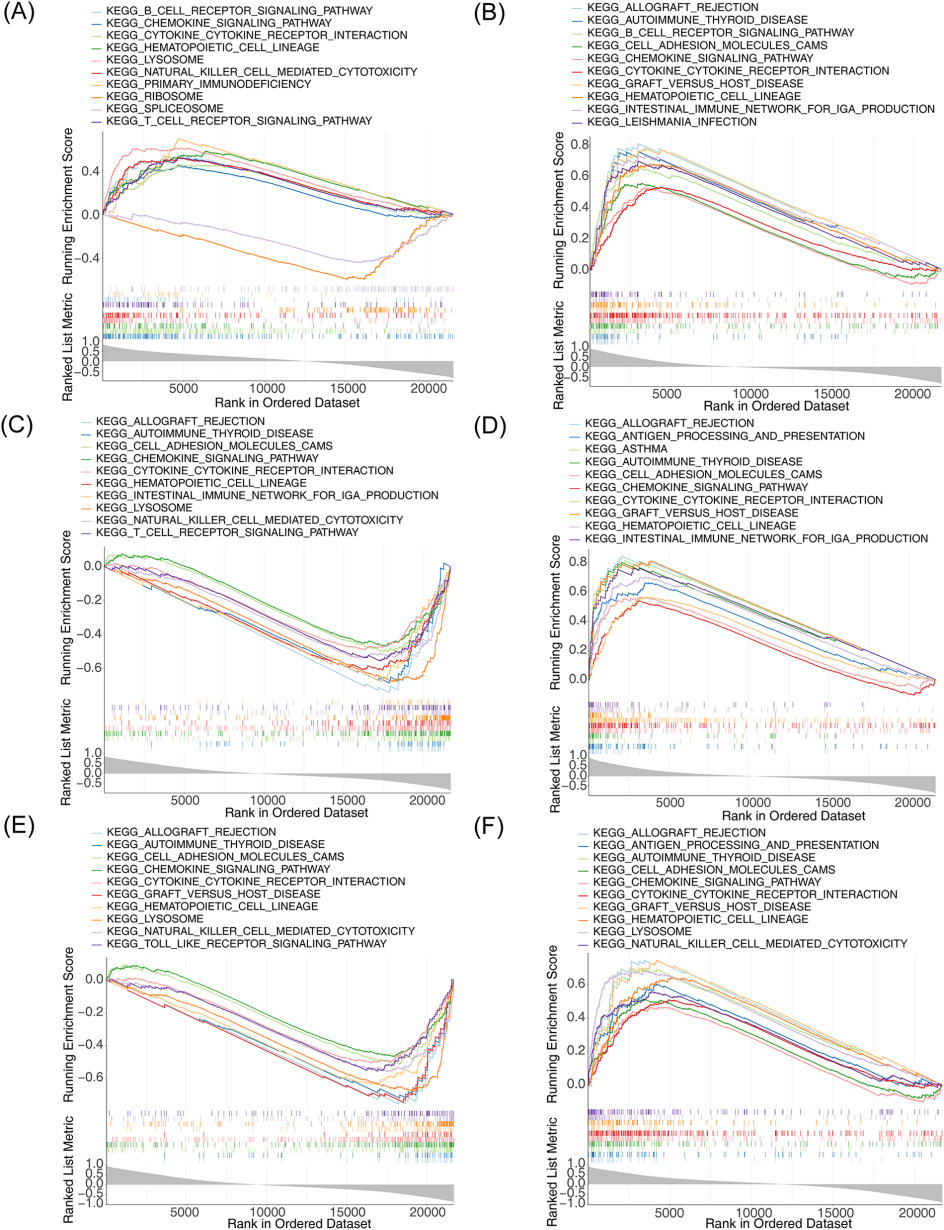
Single-gene GSEA for m6A-Ferr-related signature genes. **(A)** Results of KEGG enrichment analysis for *AGPAT3* using the single-gene GSEA method (top 10). **(B)** KEGG enrichment results for *ATG7* using the single-gene GSEA method. **(C)** KEGG enrichment results for *CDO1* using GSEA the single-gene method. **(D)** KEGG enrichment results for *CYBB* using the single-gene GSEA method. **(E)** KEGG enrichment results for *NOX4* using the single-gene GSEA method. **(F)** KEGG enrichment results for *SLC3A2* using the single-gene GSEA method.

To understand the biological function and the involved signaling pathways of the m6A-Ferr-related signature genes, GSEA was performed. The top 10 GO entries and KEGG pathways of the six genes are displayed in Figures 6A–F, 7A–F. We found that these genes were involved in B-cell-mediated immunity, activated immune response, adaptive immune response, the T-cell receptor signaling pathway, the B-cell receptor signaling pathway, and autoimmune thyroid disease among other BP and pathways (Figures 6A–F, 7A–F).

### 3.7 Immuno-infiltration analysis

**Figure 8:**
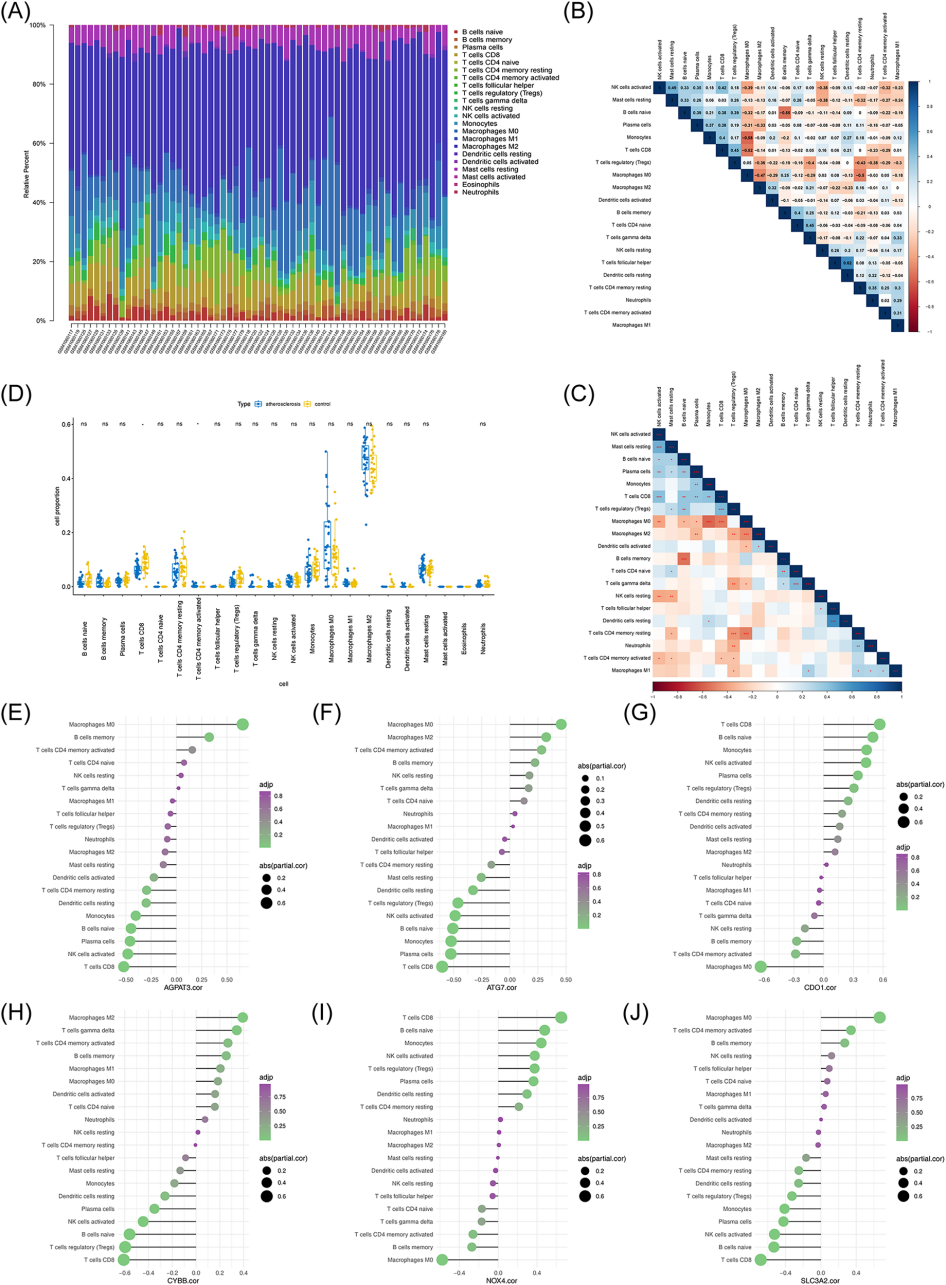
Immune infiltration analysis of atherosclerosis vs. control samples. **(A)** Ratio representation of immune infiltrating cells. **(B)** Correlation heatmap depicting proportions of immune cells. **(C)** *p*-value correlation matrix indicating significance levels among immune cell proportions. **(D)** Box plot comparing differences in immune cell content between the atherosclerosis and control groups. **(E)** Correlation analysis between immune cell content and *AGPAT3*. **(F)** Correlation analysis between immune cell content and *ATG7*. **(G)** Correlation analysis between immune cell content and *CDO1*. **(H)** Correlation analysis between immune cell content and *CYBB*. **(I)** Correlation analysis between immune cell content and *NOX4*. **(J)** Correlation analysis between immune cell content and *SLC3A2*.

Since the GSEA results suggested that the m6A-Ferr-related signature genes in AS were linked to immune-related functions, we then performed an immune infiltration analysis. By analyzing the ratio of 22 immune cells in the AS and control samples, an abundance map of immune cell infiltration proportions was constructed to demonstrate the immune cell content in the samples (Figure 8A). A correlation heat map and *p*-value correlation heat map of the immune cell ratio are shown in Figures 8B,C. A total of two types of immune cells (CD8+ T cells and activated CD4+ memory T cells) showed significant differences between the AS and control groups (Figure 8D). The correlations of the 22 immune cells with the six m6A-Ferr-related signature genes were calculated and individually plotted in lollipop plots (Figures 8E–J). According to *p*_adj_ <0.5 and |cor| >0.3, naive B cells, CD8+ T cells, regulatory T cells (Tregs), and activated natural killer (NK) cells were positively correlated with *CDO1* and *NOX4* but negatively correlated with *ATG7*, *CYBB*, and *SLC3A2*. In addition, *AGPAT3* was negatively correlated with naive B cells, CD8+ T cells, and activated NK cells, and *SLC3A2* was positively correlated with activated CD4+ memory T cells.

## 4 Discussion

AS is the primary cause of most cardiovascular events with the highest incidence rate and mortality among other causes worldwide (28). M6A modification plays an important role in various BP, including AS (29, 30). Ferroptosis is a regulated form of cell death attributed to an imbalance in the production and clearance of lipid peroxides caused by abundant cellular iron levels, which are also closely related to AS (8). It has been proven that the mRNA of various ferroptosis regulatory factors can be labeled with m6A modification. m6A participates in ferroptosis by regulating the mRNA stability, protein expression, and modification of multiple genes related to ferroptosis (15), and this has been demonstrated in the field of cancer research (31). The exploration of target genes for M6A-related ferroptosis in AS holds great potential. However, the link between M6A-related ferroptosis and AS has not yet been elucidated.

Through LASSO regression, this project screened three diagnostic genes for M6A-related ferroptosis in AS and constructed a diagnostic risk model for AS. We utilized WGCNA and LASSO analysis to identify six diagnostic genes associated with m6A-mediated ferroptosis in AS, namely *AGPAT3*, *NOX4*, *CDO1*, *CYBB*, *ATG7*, and *SLC3A2*. In addition, we also identified three m6A-related ferroptosis marker genes (*NOX4*, *CDO1*, and *SLC3A2*) using an RT-qPCR. Through the validation of expression levels, ROC mapping, single-gene GSEA, and immune infiltration analysis of these diagnostic genes, we revealed the relationship between these genes and AS at various levels. Subsequently, we constructed a diagnostic risk model for AS. This diagnostic risk model is beneficial for the clinical assessment of AS risk. Our findings demonstrate that all three m6A-Ferr-related characteristic genes have potential diagnostic value for AS. Several studies have presented evidence regarding the mechanisms and roles of these individual genes in AS. The production of ROS by *NOX4* is the molecular basis of AS, hypertension, and other chronic diseases (32). Furthermore, the active mediation of endothelial cell activation, dysfunction, and injury by *NOX4* contributes to the development of AS (33). Studies have indicated that *CDO1* may function as a co-activator of PPAR *γ* in adipogenesis, potentially contributing to the pathogenesis of diseases associated with excessive adipose tissue such as AS (34). Promoting *SLC3A2*-related endothelial cell ferroptosis leads to endothelial cell damage and progression of AS plaque (35). Interestingly, the GO enrichment pathways found in this study include ferroptosis and chemical carcinogenesis-reactive oxygen species. These findings are consistent with the results of the related research mentioned above.

The progression of AS involves intricate interactions and phenotypic plasticity between blood vessels and immune cell lineages (36). Both the congenital and adaptive immune systems play a pivotal role in driving the chronic inflammation of arteries that is related to AS (37). The immune cells involved in AS include T cells, B cells, NK cells, Natural killer T cells, macrophages, monocytes, dendritic cells (DC), neutrophils, and mast cells (38). Our research findings indicate that the m6A-Ferr-related marker genes in AS are associated with immune functions. The results of the immune infiltration analysis revealed a positive correlation between immature B cells, CD8+ T cells, Tregs, and activated NK cells with *CDO1* and *NOX4*, while they showed a negative correlation with *SLC3A2*. In addition, *SLC3A2* was positively correlated with activated CD4+ memory T cells. In a mouse model of AS, antibody-mediated depletion of CD8+ T cells led to an improvement in AS. It has been observed that CD8+ T cells play a role in controlling monogenesis and macrophage accumulation in the early stages of AS (39). In addition, CD4+ T cells are commonly found in atherosclerotic plaques. There is substantial evidence indicating that helper T cell 1 (TH1) promotes AS, while Tregs have an anti-atherosclerotic effect. The roles of other TH cell subpopulations, follicular helper T cells, and T-cell subpopulations in AS remain unclear (40). Studies have demonstrated that *CDO1* redox immune-related genes can serve as indicators of the immune microenvironment (41). ROS generate *NOX4* enzymes, which are known to play a role in immune defense (42). The downregulation of *SLC3A2* contributes to immune evasion, and targeting *SLC3A2* can regulate the metabolic adaptability of immune cells and modulate cytokine production in human plasma such as dendritic cells (pDCs) (43). Successful immunotherapy for AS requires customization to address specific immune changes in different patient groups (44). In this study, three m6A-related ferroptosis genes were identified as potential biomarkers for AS. These genes are involved in BP and pathways such as B-cell-mediated immunity, activated immune response, adaptive immune response, the T-cell receptor signaling pathway, the B-cell receptor signaling pathway, and autoimmune thyroid disease. Currently, the treatment of AS has expanded from simple lipid-lowering and plaque stabilization to implementing immune prevention and control (45). This research provides a new perspective by revealing new immune mechanisms and cell type-specific pathways in AS.

In conclusion, our study demonstrates that all three diagnostic genes exhibit strong efficacy for risk diagnosis in AS. Compared to previous single genome or epigenetic analyses, the combined predictive model, composed of three diagnostic genes for m6A and ferroptosis, more accurately reflects the progression and prognosis of AS. In addition, this study investigated the correlation between three genes and immune infiltration related to AS, laying a theoretical foundation for immunotherapy in the field of AS. However, there are limitations in our research. First, this study did not elucidate the specific mechanisms by which three m6A-related ferroptosis genes regulate AS based on LASSO; further exploration is needed through animal experiments and the collection of clinical samples. Second, the AS datasets included in this study ignored the impact of population heterogeneity in different countries on the results.

## Data Availability

The datasets presented in this study can be found in online repositories. The names of the repository/repositories and accession number(s) can be found in the article/Supplementary Material.
