# Supplementary material for: Screening of m6A-associated ferroptosis-related genes in atherosclerosis based on WGCNA
Source: Front Cardiovasc Med. 2024 Oct 28;11:1469805. doi: 10.3389/fcvm.2024.1469805 (PMC11550986; doi:10.3389/fcvm.2024.1469805)
Supplement: Supplementary file 3 [file Table3.doc]

| **primer** | **sequence** |
| --- | --- |
| AGPAT3 F | CTGAAGACCCAGTTCGTGCT |
| AGPAT3 R | CCAGTTGGCTCCAGAGTGAG |
| NOX4 F | TCTTTGACCCTCGGTCCTCG |
| NOX4 R | TTTAACCCCTTCGTTGGCGA |
| CDO1 F | GTCTCTGCGACGACATCCTT |
| CDO1 R | GGTCTGTTCCATCTCGTGGG |
| CYBB F | TGTCAAGTGCCCAAAGGTGT |
| CYBB R | CCCAACGATGCGGATATGGA |
| ATG7 F | TGTGCCTCACCAGGTTCTTGAT |
| ATG7 R | CGCTCATGTCCCAGATCTCA |
| SLC3A2 F | GGGCCTGGACTCTTCTCCTA |
| SLC3A2 R | CCTTCGTGAGGCTCCAGTTT |
| internal reference-GAPDH F | CGAAGGTGGAGTCAACGGATTT |
| internal reference-GAPDH R | ATGGGTGGAATCATATTGGAAC |
